# Insulator Umbrella Disc Shedding Detection in Foggy Weather

**DOI:** 10.3390/s22134871

**Published:** 2022-06-28

**Authors:** Rui Xin, Xi Chen, Junying Wu, Ke Yang, Xinying Wang, Yongjie Zhai

**Affiliations:** 1 State Grid Hebei Information and Telecommunication Branch, Shijiazhuang 050013, China; xinruicn@163.com (R.X.); m18830267026@163.com (X.C.); kkyy237@163.com (J.W.); 2 Department of Automation, North China Electric Power University, Baoding 071003, China; wangxinying@ncepu.edu.cn (X.W.); zhaiyongjie@ncepu.edu.cn (Y.Z.)

**Keywords:** insulator umbrella disc shedding, defect detection, dehazing algorithm, feature fusion, attention mechanism

## Abstract

The detection of insulator umbrella disc shedding is very important to the stable operation of a transmission line. In order to accomplish the accurate detection of the insulator umbrella disc shedding in foggy weather, a two-stage detection model combined with a defogging algorithm is proposed. In the dehazing stage of insulator images, solving the problem of real hazy image data is difficult; the foggy images are dehazed by the method of synthetic foggy images training and real foggy images fine-tuning. In the detection stage of umbrella disc shedding, a small object detection algorithm named FA-SSD is proposed to solve the problem of the umbrella disc shedding occupying only a small proportion of an aerial image. On the one hand, the shallow feature information and deep feature information are fused to improve the feature extraction ability of small targets; on the other hand, the attention mechanism is introduced to strengthen the feature extraction network’s attention to the details of small targets and improve the model’s ability to detect the umbrella disc shedding. The experimental results show that our model can accurately detect the insulator umbrella disc shedding defect in the foggy image; the accuracy of the defect detection is 0.925, and the recall is 0.841. Compared with the original model, it improved by 5.9% and 8.6%, respectively.

## 1. Introduction

**Figure 1:**
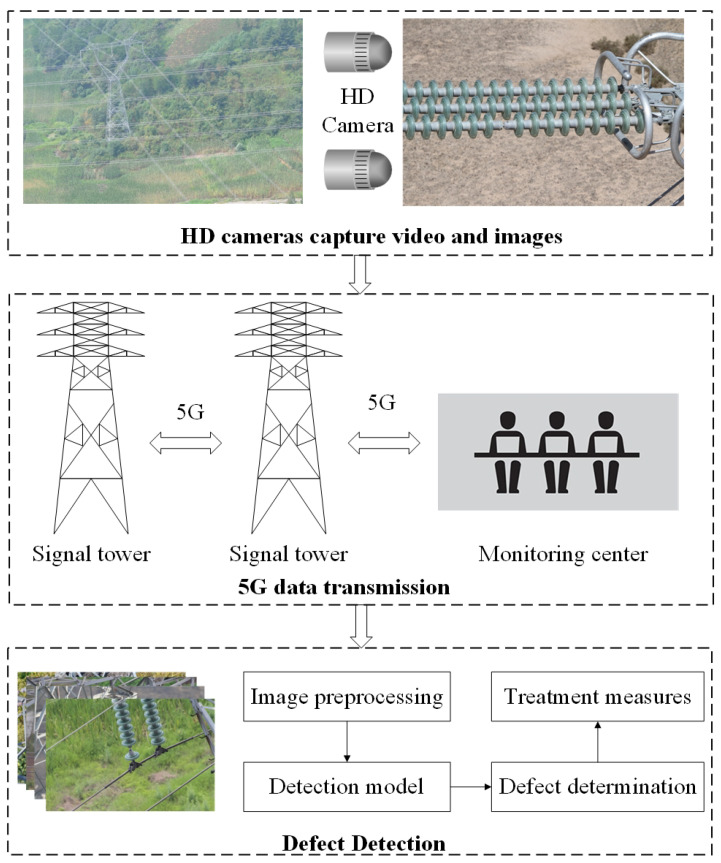
Insulator defect detection based on 5G and AI.

Transmission lines are an important part of the power grid and are crucial to the safe and stable operation of the power grid [1,2]. In transmission lines, insulators are the basic equipment used for electrical isolation and mechanical fixation in high-voltage transmission systems [3,4]. Since the insulators remain exposed, environmental factors inevitably cause damage to them, and the resulting insulator failures can seriously affect the safe and stable operation of the power grid [5,6]. Therefore, timely detection of insulator defects and early treatment can effectively reduce the occurrence of insulator failures [7,8]. The defects of insulators mainly include umbrella disc shedding, umbrella disc damage, dirt, and icing. Among these defects, insulator umbrella disc shedding is the most common, the most numerous, and the most harmful defect. With the rapid development of 5G technology and AI technology [9,10], combined with 5G high-speed data transmission and target detection technology, through the all-weather monitoring of transmission lines, insulator defects can be found in time, effectively reducing the transmission line failures caused by insulator defects [11]. The advantage of 5G technology is that it can achieve high-speed data transmission, which can not only ensure image quality but also ensure real-time detection. Transmission line insulator defect detection based on 5G and AI is shown in Figure 1. First, HD cameras take videos of insulators; second, the captured data are compressed and transmitted to the monitoring center through 5G communication; third, stsffs decompress the data, process the video frame by frame, and use the corresponding defect detection model to detect and judge the defect level; finally, technicians take corrective measures, according to the defect level.

At present, the mainstream dehazing algorithms mainly include the dehazing algorithm based on image enhancement, the dehazing algorithm based on image restoration, and the dehazing algorithm based on CNN. The first method uses image processing to highlight image details and enhance contrast to make foggy images clearer. The specific algorithms include histogram equalization [12], wavelet transform [13], and the Retinex algorithm [14]. The second method is based on the physical model of atmospheric scattering, which can obtain the mapping relationship between the foggy image and the fog-free image; and it restores the foggy image to a clear image. The most representative algorithm is the dark channel prior dehazing algorithm proposed by He [15]. However, physical priors are not always reliable, and these priors do not apply to all hazy images, which makes the dehazing effect uncertain. The third method builds an end-to-end model through CNN to recover clear images from hazy images [16,17]. Such methods overcome the disadvantage of using physical priors; they are more efficient and perform better than traditional prior-based algorithms. Zhao [18] proposed a novel end-to-end convolutional neural network called the attention enhanced serial Unet++ [19] dehazing network (AESUnet) for single image dehazing, and the serial Unet++ module generated more realistic images with less color distortion. Gao [20] proposed an image dehazing model built with a convolutional neural network and Transformer to improve the quality of the restored image. However, CNN requires a large number of hazy and clear image pairs for training, which are difficult to obtain. Due to the lack of real foggy image datasets, many studies are carried out on synthetic foggy images, which makes it difficult to achieve good results when the dehazing algorithms are applied to real foggy images.

**Figure 2:**
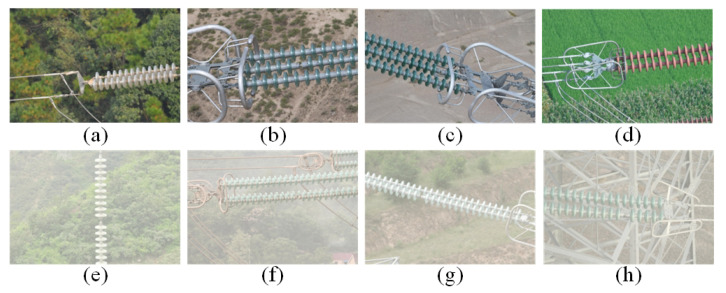
Images of insulators in foggy and fog-free weather conditions. (**a**–**d**) Clear images. (**e**–**h**) Foggy images.

Researchers have investigated insulator defect detection. Zhang [21] proposed an optical image detection method based on deep learning and morphological detection. First of all, the Faster RCNN was used to locate the insulator and extract its target image from the detection image. Second, a segmentation method of the insulator image was proposed to remove the background of the target image. Finally, a mathematical model was established in the binary image to describe the defect of the insulator. Tao [22] proposed a novel deep CNN cascaded architecture to perform localization and detection of defects in insulators. The cascaded network transformed defect detection into a two-level object detection problem, which used a region proposal network-based CNN. The method first detected the insulator in the aerial image and then detected the shedding defect of the insulator umbrella disk on this basis. She [23] proposed a multiscale residual neural network for insulator surface damage identification, using three convolution kernels of different sizes to perform convolution filtering and feature map fusion to enrich the spatial correlation and channel correlation of feature maps. Aiming at the small proportion of the insulator umbrella disc shedding fault area in the entire image and the difficulty in detection, Zahng [24] introduced the densely connected feature pyramid network into the YOLOV3 [25] model to achieve high detection accuracy. Zhao [26] combined Faster R-CNN [27] and an improved FPN [28] to detect two types of insulator defects. However, the above studies were all to detect insulator defects under normal weather conditions. Under real environmental conditions, one will inevitably encounter complex weather conditions [29,30,31]. Foggy weather is the most common complex weather. Achieving the detection of insulator defects in foggy conditions is crucial for all-weather real-time monitoring of transmission lines. As shown in Figure 2, there is a clear difference between the insulator images in foggy and fog-free weather conditions.

This paper proposes a detection method for insulator umbrella disc shedding in foggy weather conditions. The main contributions of this paper are as follows:

(1) For the first time, the detection of insulator umbrella disc shedding in foggy conditions is realized, which provides a new way to detect transmission line defects in complex weather.
(2) A dehazing model with synthetic image pre-training and real image fine-tuning is proposed to solve the problem of the poor dehazing effect on real hazy images.
(3) The FA-SSD model [32] is proposed to improve the accuracy and recall rate of insulator umbrella disc shedding detection.

## 2. Materials and Methods

**Figure 3:**
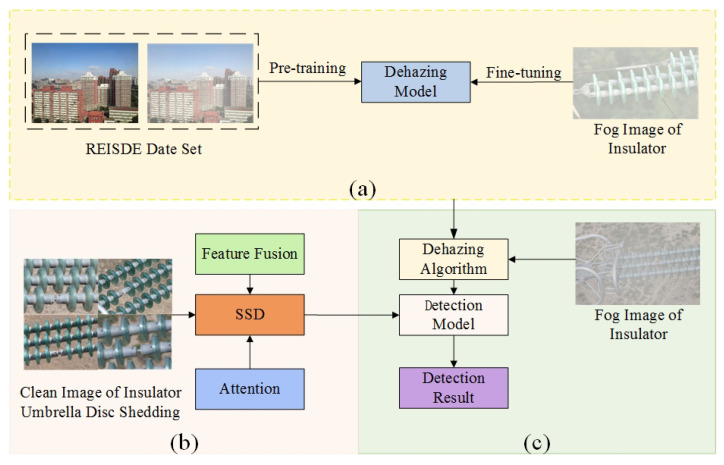
The overall process of umbrella disc shedding detection. (**a**) Dehaze model. (**b**) Training phase. (**c**) Testing phase.

As shown in Figure 3, the overall process of umbrella disc shedding detection included three parts: pre-training and fine-tuning of the defogging model, training with the clear insulator image datasets, and testing with the fogged insulator images.

The dehazing model was trained by synthetic foggy images, and the insulators with foggy images were fine-tuned to improve the dehazing effect of the algorithm. A feature fusion module and an attention module were added to the umbrella disc shedding detection model to improve the detection accuracy. In the detection of the insulator umbrella disc shedding, clear images of insulators were used for training, and images of insulators with fog were used for testing.

### 2.1. Dehazing Model

**Figure 4:**
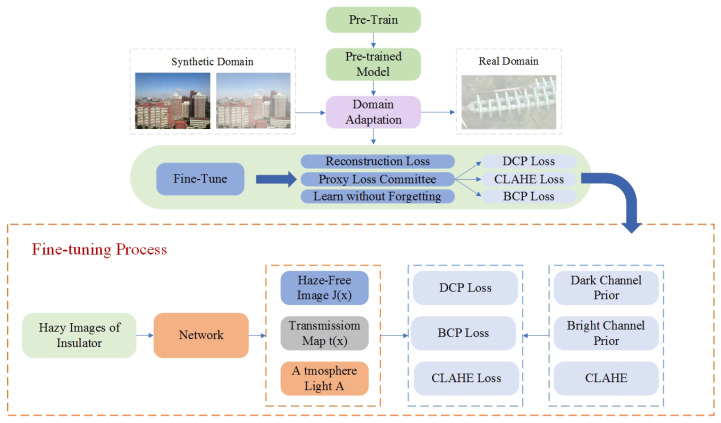
Structure of the dehaze model.

Inspired by the dehazing algorithm proposed by Chen [33], this paper adopted the method of pre-training and fine-tuning to improve the dehazing effect of the dehazing model. The training of the model was divided into two steps. The first step used a large number of haze-free images and artificially-generated fogged images from the REISDE dataset [34] to train the dehazing model, and the second step used the foggy insulator images to fine-tune the dehazing model to improve the dehazing ability of the dehazing model on fogged insulator images. During fine-tuning, physical priors were guided through the loss function. As shown in Figure 4, the dehazing model had a two-stage framework.

In the pre-training stage, an advanced dehazing model was adopted as the backbone. The pre-training phase used synthetic data for training, resulting in a pre-trained model on the synthetic domain. In the fine-tuning stage, the fog-free image *J*, transmission map *t*, and atmospheric light *A* were obtained through the backbone network. At the same time, three priors, including a dark channel prior, a bright channel prior, and the Contrast Limited Adaptive Histogram Equalization (CLAHE) were introduced, and the model was guided in the form of loss function.

The loss function of the dark channel prior is shown as follows:

(1) $$L_{DCP}=E\left(t,\tilde{t}\right)=t^{T}Lt+\lambda {\left(t-\tilde{t}\right)}^{T}\left(t-\tilde{t}\right)$$

where *t* and $\tilde{t}$ denote the transmission estimates from the DCP and the backbone network, respectively. *L* is a Laplacian-like matrix.

The loss function of the bright channel prior is shown as follows:

(2) $$L_{BCP}={∥t-\tilde{t}∥}_{1}$$

where *t* and $\tilde{t}$ represent the transmission estimates from the BCP and the backbone network, respectively.

The loss function of the CLAHE reconstruction is shown as follows:

(3) $$L_{CLAHE}={∥I-I_{CLAHE}∥}_{1}$$

where *I* is the original hazy input, and $I_{C}LAHE$ is the reconstruction result by $J_{C}LAHE$, $\tilde{t}$, and $\tilde{A}$.

The role of the three loss functions is different. Dark channel prior greatly advances the model performance on real hazy images, bright channel prior helps make the resulting images brighter and with enhanced contrast, and CLAHE is used to achieve a balance between $L_{D}CP$ and LBCP.

The total loss of the fine-tuning process was obtained by combining the three losses as follows:

(4) $$L_{com}=\lambda_{d}L_{DCP}+\lambda_{b}L_{BCP}+\lambda_{c}L_{CLAHE}$$

where $\lambda_{d}$, $\lambda_{b}$, and $\lambda_{c}$ are the tradeoff weights.

### 2.2. Fa-Ssd Model

**Figure 5:**
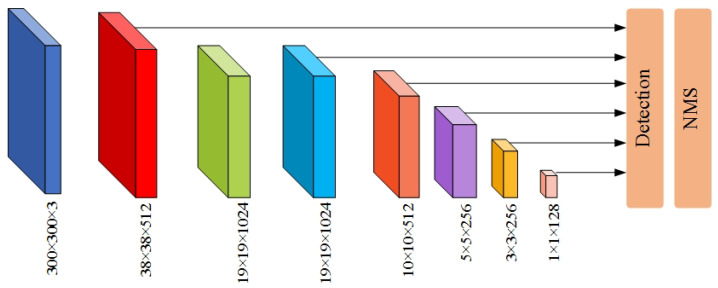
Structure of the SSD model.

Target detection includes target recognition and localization. For CNN, the two are contradictory [35]. Generally speaking, deep feature maps contain more semantic information, which is good for object recognition but not good for object localization; the difference is that the shallow feature map contains more detailed features, which is good for object localization but not good for object recognition. As shown in Figure 5, the SSD model adopts a feature pyramid structure to detect objects of different scales; small objects are detected on the shallow feature maps, and large objects are detected on the deep feature maps.

**Figure 6:**
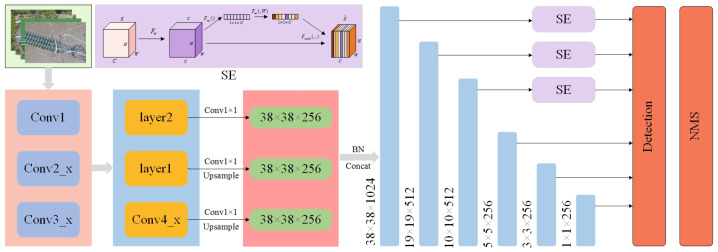
Structure of the FA-SSD model.

However, the problem with this method is that the small target features generated by the shallow layer lack sufficient semantic information, and the detection of small targets still is not effective. In order to improve the detection ability of the SSD model for the insulator umbrella disk shedding, the FA-SSD model is proposed. As shown in Figure 6, the FA-SSD model adds a feature fusion module and an attention module to the SSD model.

First, the insulator images were sent to the ResNet50 [36] feature extraction network to extract the features. Since the shallow feature maps contain richer small target detail information, Conv4_x in ResNet50 and two auxiliary convolutional layers were selected for feature fusion. The feature dimension of Conv4_x was 38 × 38 × 1024, and the feature dimensions of the two auxiliary convolutional layers were 19 × 19 × 512 and 10 × 10 × 512. Then, in order to fuse the features of the three different scales simply and efficiently, the two auxiliary convolutional layers were upsampled using bilinear interpolation to make them the same size as Conv4_x. Finally, the feature map was concatenated and normalized to generate a new feature pyramid structure for the identification and localization of umbrella disc shedding. The parameters of each layer in the structure are shown in Table 1.

On this basis, in order to enhance the network’s ability to extract low-level detail features, the SE channel attention module [37] was added to the lowest three layers of the feature pyramid.

#### SE Module

The SE learns a set of weight coefficients through a small fully connected network to weigh each channel of the original feature map. In this way, different weights are assigned to each channel to enhance the feature extraction capability of the network. The implementation process of the SE was as follows:

(1) We performed convolution pooling and other operations on the input image to obtain a feature map:
  (5) $$u_{c}=v_{c}*X=\sum_{s=1}^{c^{{}^{\prime }}}v_{c}^{s}*x^{s}$$
  where $v_{c}$ and *X* represent the convolution kernel and the input image, respectively; $v_{c}^{s}$ and $x^{s}$ represent the convolution kernel and the *s*th channel of the input image, respectively; and $c^{{}^{\prime }}$ represents the number of channels.
(2) We squeezed and compressed the feature map into one-dimensional features:
  (6) $$z_{c}=F_{sq}\left(u_{c}\right)=\frac{1}{H\times W}\sum_{i=1}^{H}\sum_{j=1}^{W}u_{c}\left(i,j\right)$$
  where *H* and *W* represent the width and height of the feature map, respectively.
(3) For excitation, we performed activation operations on multiple channels to extract different features:
  (7) $$s=F_{ex}\left(z,W\right)=\sigma \left(g\left(z,W\right)\right)=\sigma \left(W_{2}\delta \left(W_{1}z\right)\right)$$
(4) We multiplied the obtained weight factor with the corresponding channel feature to obtain a new feature map:
  (8) $$\tilde{x_{c}}=F_{scale}\left(u_{c},s_{c}\right)=s_{c}\cdot u_{c}.$$

## 3. Results

### 3.1. Experimental Environment

The proposed model used an NVIDIA RTX 2080Ti GPU for training and testing and the Ubuntu 18.04 LTS as the operating system; the training process was accelerated by CUDA 10.1; the computer language was Python 3.6, and the network framework was PyTorch. The batch size was set to 8, the learning rate was 0.003, the preprocessed size of the input image was 300 × 300, and the maximum number of iterations was 7800. The SSD was chosen as the baseline for improvement and comparison purposes.

**Figure 7:**
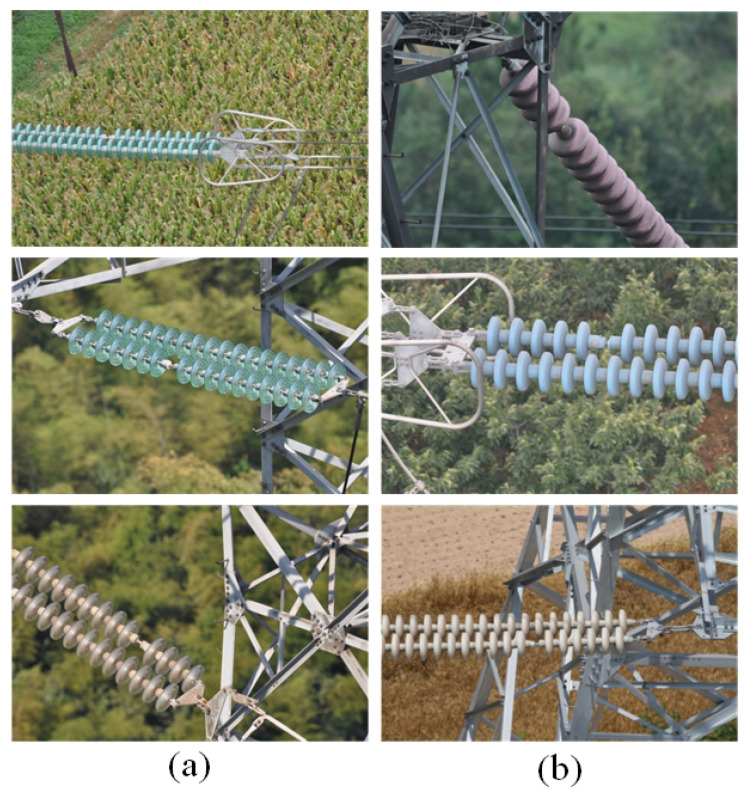
Glass insulators and ceramic insulators. (**a**) Glass insulators. (**b**) Ceramic insulators.

The datasets used in the dehazing stage included the REISDE dataset and images of fogged insulators. The insulator images used in this paper were the aerial images of transmission line inspection, which were obtained by UAV. The datasets used in the object detection stage consisted of fogged insulator images, as well as fog-free insulator images. Since the insulators were in normal working condition most of the time, the defect images occupied a small proportion of the obtained aerial images. In addition, due to factors such as shooting environment, shooting angle, shooting distance, etc., many images were of poor quality. By cooperating with several power grid companies, we obtained some samples of insulator umbrella disk shedding. Among them, there were 160 images (the number of the insulator umbrella disc shedding was 176) with fog and 480 images (the number of the insulator umbrella disc shedding was 518) without fog. We used the images without fog as the training set and the images with fog as the test set. As shown in Figure 7, the insulator datasets contained glass insulators and ceramic insulators.

To compare the different models, precision(*P*), recall(*R*), and $F_{1}$ were used as model evaluation metrics. The higher the value, the better the detection performance of the model.

(9) $$P=\frac{T_{P}}{T_{P}+F_{P}}$$

(10) $$R=\frac{T_{P}}{T_{P}+F_{N}}$$

(11) $$F_{1}=\frac{2\times P\times R}{P+R}$$

where *TP* and *FP* denote the number of correctly and incorrectly located defects, respectively. *TP* + *FP* is the total number of located defects, and *TP* + *FN* is the total number of actual defects. $F_{1}$ is the harmonic mean of precision and recall.

### 3.2. Ablation Experiment of Fa-Ssd Model

**Figure 8:**
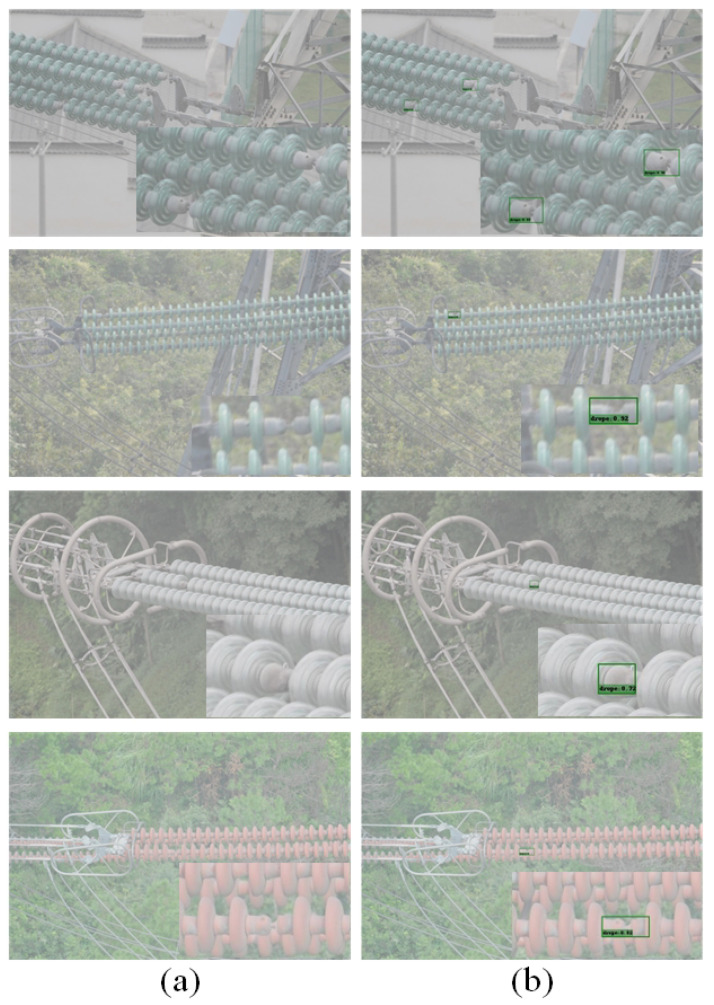
Visualization of SSD and FA-SSD. (**a**) SSD. (**b**) FA-SSD.

In order to verify the effectiveness of the feature fusion module and the attention module, the experiments were conducted on the original SSD model, the SSD model with the feature fusion module, the SSD model with the attention module, and the FA-SSD model. The visualization results of the FA-SSD model and the SSD model are shown in Figure 8.

In the experiment, the other parameters of the model training were guaranteed to be the same, and the obtained detection results are shown in Table 2.

The detection performance of the FA-SSD was better than the methods that only added the feature fusion module or the attention module. Compared with the original SSD model, the accuracy rate was improved, the recall rate was improved, and the F1 indicator was improved. The experimental results showed that both the feature fusion module and the attention module had a positive effect on the model.

### 3.3. Compared with Other Methods

**Figure 9:**
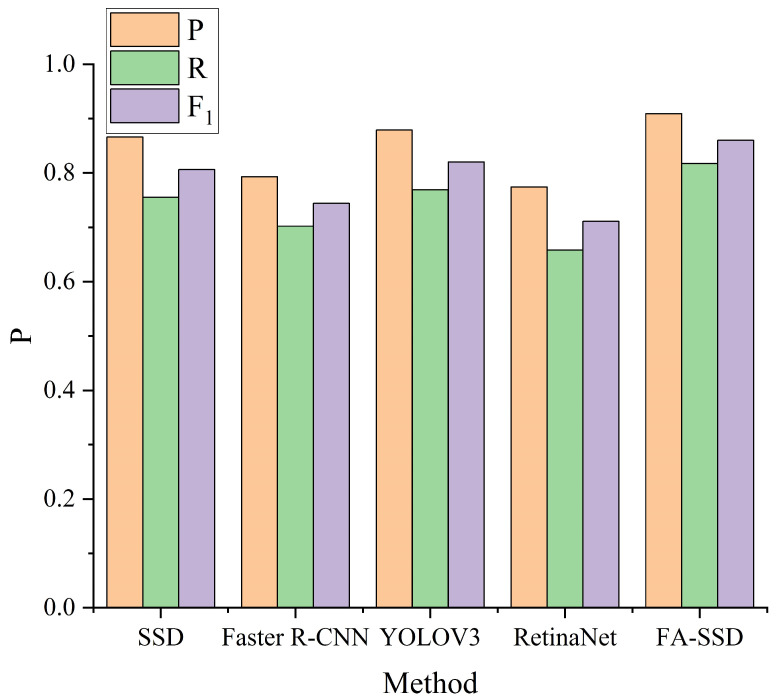
Results of different methods.

**Figure 10:**
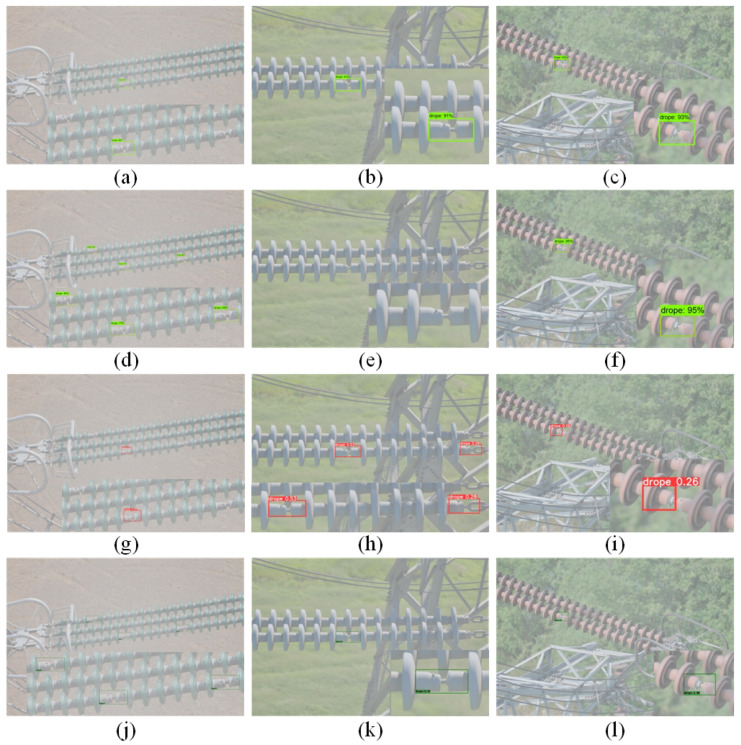
Visualization results of different methods. (**a**–**c**) Faster R-CNN. (**d**–**f**) YOLOV3. (**g**–**i**) RetinaNet. (**j**–**l**) FA-SSD.

In order to further verify the effectiveness of the FA-SSD model in the detection of insulator umbrella disk shedding, under the condition of ensuring the same feature extraction network and hyperparameters, the method in this paper was compared with the commonly used target detection algorithm at this stage. The compared methods included Faster R-CNN [27], YOLOV3 [25], and RetinaNet [38], and the results are shown in Figure 9 and Figure 10 and Table 3.

It can be seen that FA-SSD significantly outperformed SSD and other commonly used object detection algorithms. Compared with other algorithms, the accuracy rate of detecting the umbrella disc shedding was improved on average 8.1%, and the recall rate was improved on average 9.6%. Compared with other target detection algorithms, the FA-SSD algorithm improved the detection accuracy and reduced the missed detection rate.

### 3.4. Dehazing Algorithm Experiment

**Figure 11:**
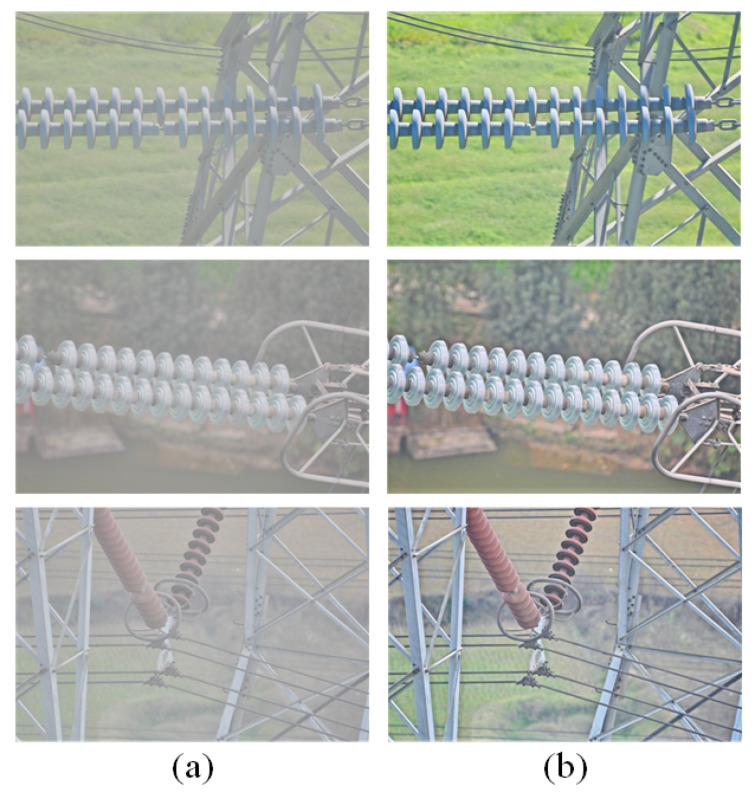
Visualization of Dehazing Algorithms. (**a**) Foggy images. (**b**) Images after dehazing.

As shown in Figure 11, after using the dehazing algorithm to dehaze the hazy images, the pictures became clearer.

**Figure 12:**
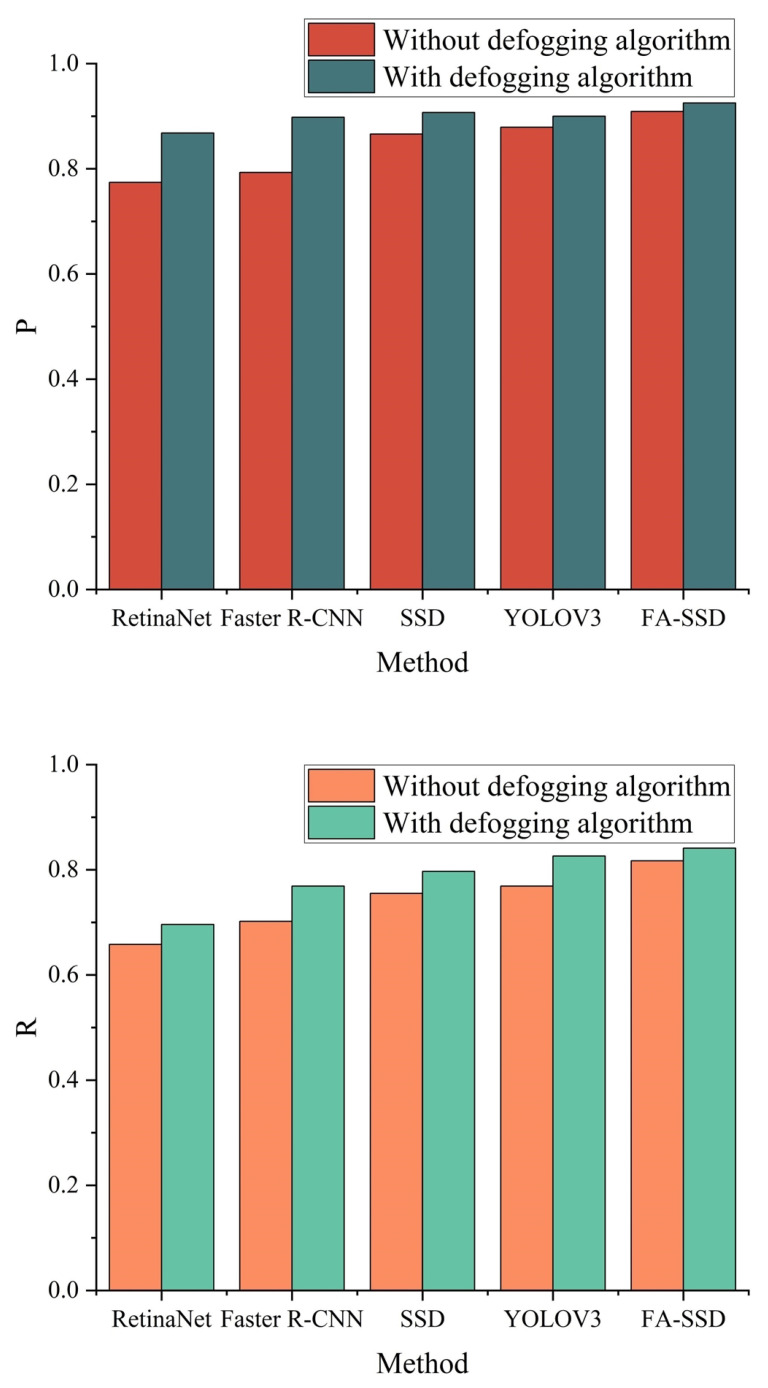
Experimental results before and after adding the defogging algorithm.

In order to verify the effectiveness of the dehazing algorithm proposed in this paper for the detection of insulator umbrella disc shedding in foggy images, the dehazing algorithm proposed in this paper was combined with the target detection algorithm, and the obtained detection results are shown in Figure 12.

**Figure 13:**
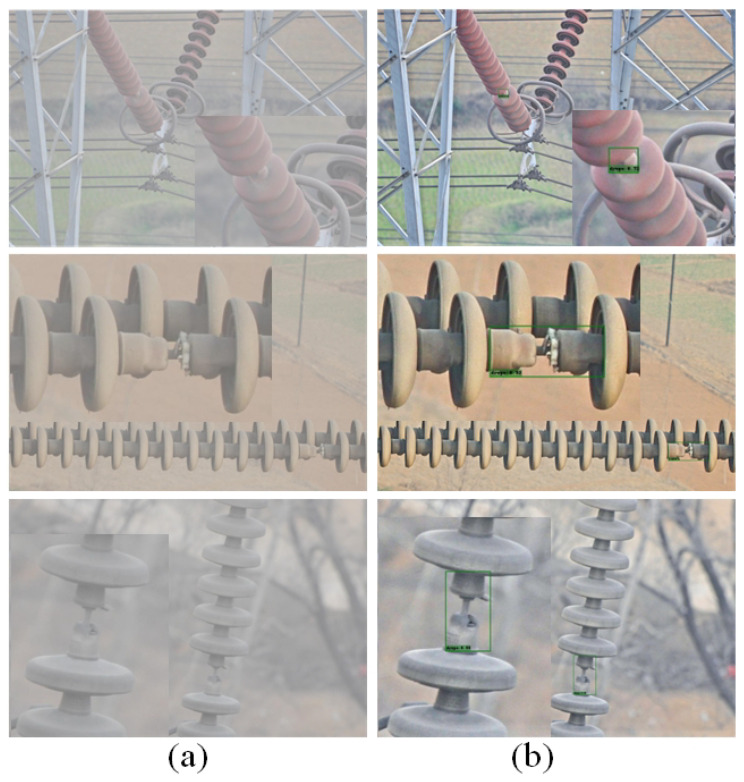
Visualization results of the FA-SSD and FA-SSD with defogging algorithm. (**a**) FA-SSD. (**b**) FA-SSD with defogging algorithm.

As shown in Figure 12, the accuracy and recall of the model proposed in this paper were better than other models. It can be seen that after adding the defogging model, the accuracy and recall rate of the insulator umbrella disc shedding detection of the other models were significantly improved. Among them, the accuracy rate of the model increased by 0.08 on average, and the recall rate increased by 0.06 on average. This is because the clear image obtained by the dehazing algorithm was more conducive to the extraction of the features, thereby improving the detection effect. As shown in Figure 13, after adding the defogging algorithm, the detection effect of the FA-SSD model was significantly improved.

## 4. Discussion

On the basis of realizing the defect detection of insulators with foggy images, combined with the high-speed transmission advantages of 5G technology, real-time detection of insulator defects can be realized, and the necessary processing methods can be taken in time to reduce insulator failures. Compared with the traditional manual inspection, the method in this paper can reduce labor, material resources, and the influence of subjective factors; compared with the currently used UAV inspection, the method in this paper is more in real time. In the context of China’s vigorous promotion of a smart grid, this research has important practical significance and good development prospects.

In the future, our research will have the following three aspects. First, we will examine more dehazing algorithms, such as the latest semi-supervised [39] or unsupervised [40] frameworks. Second, we will collect more fogged images of insulators and conduct a joint training strategy to combine image dehazing with defect detection [41]. Third, we will study the defect detection of insulators under a series of complex weather conditions such as sand, rain, and snow and devote ourselves to solving the problem of the defect detection of transmission lines in complex weather, so as to realize all-weather real-time monitoring of transmission lines.

## 5. Conclusions

Aiming to solve the difficulty of fully extracting effective features from foggy insulator images, as well as the small and difficult to detect proportion of umbrella disk shedding in an image, this paper proposed a detection method for insulator umbrella disk shedding defects that combined a dehazing algorithm and FA-SSD. Through the two-stage algorithm of dehazing and detection, the accurate detection of the insulator umbrella disk shedding in a foggy image was realized. This paper is the first to detect the defects in transmission lines with foggy images, which provides a solution for all-weather monitoring of transmission lines under complex weather conditions.

## Figures and Tables

**Table 1:** Input and output dimensions of each layer.

Layer Name	Input	Output
Conv1	300 × 300 × 3	150 × 150 × 64
Conv2_x	150 × 150 × 64	75 × 75 × 256
Conv3_x	75 × 75 × 256	38 × 38 × 512
Conv4_x	38 × 38 × 512	38 × 38 × 1024
layer1	38 × 38 × 1024	19 × 19 × 512
layer2	19 × 19 × 512	10 × 10 × 512

**Table 2:** Results of the ablation experiment.

SSD	Feature Fusion	Attention	*P*	*R*	$F_{1}$
√			0.866	0.755	0.806
√	√		0.899	0.769	0.828
√		√	0.877	0.793	0.832
√	√	√	**0.909**	**0.817**	**0.860**

**Table 3:** Results of different methods.

Method	Input Size	*P*	*R*	$F_{1}$
SSD [32]	300 × 300	0.866	0.755	0.806
Faster R-CNN [27]	800 × 800	0.793	0.702	0.744
YOLOV3 [25]	300 × 300	0.879	0.769	0.820
RetinaNet [38]	300 × 300	0.774	0.658	0.711
FA-SSD	300 × 300	**0.909**	**0.817**	**0.860**

## Data Availability

Not applicable.

## Abbreviations

The following abbreviations are used in this manuscript:

5G	Fifth Generation Mobile Communication Technology
AI	Artificial Intelligence
HD	High Definition
SSD	Single Shot MultiBox Detector
NMS	Non-maximum suppression
YOLO	You Only Look Once
CNN	Convolutional Neural Network
FA-SSD	SSD Combining Feature Fusion and Attention Mechanism
FPN	Feature Pyramid Network
AR	Augmented Reality
GPU	Graphics Processing Unit
P	Precision
R	Recall
TP	True Positive
FP	False Positive
FN	False Negative
UAV	Unmanned Aerial Vehicle
